# Mechanism of antibacterial resistance, strategies and next-generation antimicrobials to contain antimicrobial resistance: a review

**DOI:** 10.3389/fphar.2024.1444781

**Published:** 2024-08-16

**Authors:** Wubetu Yihunie Belay, Melese Getachew, Bantayehu Addis Tegegne, Zigale Hibstu Teffera, Abebe Dagne, Tirsit Ketsela Zeleke, Rahel Belete Abebe, Abebaw Abie Gedif, Abebe Fenta, Getasew Yirdaw, Adane Tilahun, Yibeltal Aschale

**Affiliations:** ^1^ Department of Pharmacy, College of Health Sciences, Debre Markos University, Debre Markos, Ethiopia; ^2^ Department of Medical Laboratory Science, College of Health Sciences, Debre Markos University, Debre Markos, Ethiopia; ^3^ Department of clinical pharmacy, College of medicine and health sciences, University of Gondar, Gondar, Ethiopia; ^4^ Department of environmental health science, College of Health Sciences, Debre Markos University, Debre Markos, Ethiopia

**Keywords:** modification of target, decreased accumulation, enzymatic inactivation, biofilm formation, horizontal gene transfer, next-generation antimicrobials

## Abstract

Antibacterial drug resistance poses a significant challenge to modern healthcare systems, threatening our ability to effectively treat bacterial infections. This review aims to provide a comprehensive overview of the types and mechanisms of antibacterial drug resistance. To achieve this aim, a thorough literature search was conducted to identify key studies and reviews on antibacterial resistance mechanisms, strategies and next-generation antimicrobials to contain antimicrobial resistance. In this review, types of resistance and major mechanisms of antibacterial resistance with examples including target site modifications, decreased influx, increased efflux pumps, and enzymatic inactivation of antibacterials has been discussed. Moreover, biofilm formation, and horizontal gene transfer methods has also been included. Furthermore, measures (interventions) taken to control antimicrobial resistance and next-generation antimicrobials have been discussed in detail. Overall, this review provides valuable insights into the diverse mechanisms employed by bacteria to resist the effects of antibacterial drugs, with the aim of informing future research and guiding antimicrobial stewardship efforts.

## Introduction

Understanding the types and mechanisms of antibacterial resistance allows for the development of more effective treatment strategies against resistant pathogens. By comprehending how bacteria evade the effects of antibacterials, researchers can design novel drugs that target these resistance mechanisms, thus enhancing treatment outcomes. In addition, it enables the surveillance and monitoring of resistant bacteria, aiding in the identification of emerging threats and the implementation of appropriate infection control measures. Furthermore, knowledge of resistance mechanisms can guide antimicrobial stewardship efforts, promoting the judicious use of antibacterials to mitigate the development and spread of resistance.

Although Antimicrobial resistance (AMR) has been found in viruses, parasites, fungi, and bacteria (Prestinaci et al., 2015), antibacterial resistance stands out as a critical global public health and socioeconomic issue. Hence, this review briefly explained the types and mechanisms of antibacterial resistance. In addition, biofilm formation and horizontal gene transfer (HGT) methods have been discussed. Moreover, it overviews the strategies and next-generation antimicrobials to contain antimicrobial resistance. In this review, the term “antibacterial” refers to drugs, whether synthetic or natural, that are used to treat bacterial infections by killing bacteria or inhibiting their growth.

## Types of antibacterial drug resistance

The presence of bacteriostatic or bactericidal antimicrobial agents can enable the growth of resistant microorganisms at concentrations that normally inhibit their growth. This resistance often arises from mutations or the transfer of genetic elements that are resistant to antibacterials (acquired resistance). However, resistance can also be intrinsic, relying on the cell’s inherent characteristics and wild-type genes (Cox and Wright, 2013; Blair et al., 2015). Drug resistance can be classified into three categories: intrinsic resistance, acquired resistance, and adaptive resistance, which are determined by the way of resistance development (Lee, 2019; Bo et al., 2024a). Different literature sources may not consistently classify types of drug resistance in the same manner. Nonetheless, categorizing drug resistance as natural, acquired, and adaptive appears to be a suitable approach.

### Natural resistance

Natural resistance can either be intrinsic (always present in the species) or induced (where genes naturally found in the bacteria are only expressed to resistance levels after antibacterial exposure). Intrinsic resistance is a characteristic universally shared within a bacterial species, independent of prior antibacterial exposure, and unrelated to HGT (Cox and Wright, 2013; Martinez, 2014; Uddin et al., 2021). Intrinsic antimicrobial resistance is a microorganism’s inherent trait that makes it resistant to a particular antibacterial. Consequently, treatment with that antibacterial will be ineffective (Aarestrup, 2006; Melander et al., 2023). The most common bacterial mechanisms contributing to intrinsic resistance include reduced permeability of the outer membrane, particularly due to lipopolysaccharides (LPS) in Gram-negative bacteria, and the natural activity of efflux pumps. Additionally, multidrug-efflux pumps are a frequent mechanism behind induced resistance (Fajardo et al., 2008; Cox and Wright, 2013). Despite natural resistance is classified as intrinsic and induced resistance, genes are naturally found in the bacteria in both resistance sub-types, which differentiates it from acquired resistance. Moreover, natural resistance is primarily intrinsic.

Intrinsic resistance results from the structural characteristics of bacteria and is not related to antibacterial use. For instance, cell wall-less bacteria like Mycoplasma and Ureaplasma are naturally resistant to beta-lactam antibacterials, which target cell wall synthesis (Jawetz et al., 1995; Mayer et al., 1995; Nikaido, 2009). One example of intrinsic resistance in Gram-negative bacteria is the alteration of the glycopeptide in the bacterial cell envelope, which increases the impermeability of the outer membrane (Christaki et al., 2020). Furthermore, the restriction of antibacterial entry, caused by porin proteins, contributes to antibacterial resistance (Bellido et al., 1992). Examples of natural antibacterial resistance include: Anaerobic bacteria are resistant to aminoglycosides due to no oxidative metabolism for uptake of antibacterial (Ramirez and Tolmasky, 2010); Aerobic bacteria are resistant to metronidazole due to inability to reduce drug to its active form (Hecht, 2004) and Gram-negative bacteria are resistant to vancomycin due to impermeability of the outer membrane to large glycopeptides (Miller et al., 2014). In intrinsic resistance, antibacterial drugs were initially ineffective in treating infections caused by these bacteria. Therefore, intrinsic resistance is an inherent characteristic of the bacteria and does not present a significant challenge.

### Acquired resistance

Acquired resistance refers to the resistance that occurs when a bacterium, previously sensitive to an antibacterial, develops resistance through either a mutation or the acquisition of new genetic material from an external source via HGT (Verraes et al., 2013; Holmes et al., 2016; Munita and Arias, 2016; Durao et al., 2018; Uddin et al., 2021; Gonzalez-Villarreal et al., 2022). It arises from changes, such as mutations, in the structures of the chromosome or extrachromosomal elements like plasmids or transposons (Salih Cesur Ali and Demiröz, 2013).

Chromosomal resistance originates from mutations that spontaneously arise in the bacterial chromosome. These mutations can occur due to various physical (such as ultraviolet radiation) and chemical factors, which induce structural changes in bacterial cells. This can result in reduced permeability to drugs or alterations in drug targets within the cell. The rate of spontaneous chromosomal mutations is very low, and as a result, clinically significant resistance from this mechanism is rare and often inconsequential (Jawetz et al., 1995; Yüce, 2001). Extrachromosomal resistance relies on extrachromosomal genetic elements, which can be transferred through mechanisms such as plasmids, transposons, and integrons. Plasmid genes often encode enzymes that render antibacterials inactive. antibacterial resistance genes, whether on the chromosome or plasmid, are often interconnected and located proximally to specific integration sites, forming what are known as integrons (Jawetz et al., 1995; Mayer et al., 1995; Yüce, 2001). As a result, extrachromosomal resistance is generally more concerning because it can spread more easily and rapidly across different bacterial populations, increasing the risk of multidrug-resistant infections that are harder to treat and control. In summary, acquired resistance arises from either genetic mutations or the acquisition of new genetic material through HGT.

### Adaptive drug resistance

Adaptive drug resistance is characterized as the capacity of microorganisms to adapt reversibility and become resistant to one or more antibacterials in response to specific environmental signals. The drug resistance response to some environmental conditions, such as stress, growth state, pH, concentrations of ions, nutrient conditions, or exposure to sub-inhibitory levels of antibacterials. Adaptive drug resistance is temporary in nature, which is different from other types of drug resistance (Sandoval-Motta and Aldana, 2016; D’Aquila et al., 2023). It enables bacteria to respond swiftly to antibacterial challenges, but once the inducing signal is no longer present, the bacteria typically revert to their original susceptibility to the antibacterials (Lazar et al., 2023).

Meanwhile, the above types of drug resistance, especially acquired drug resistance may cause cross resistance, multi-drug resistance, extensive drug resistance and pan-drug resistance.

### Cross resistance

Some microorganisms that are resistant to a specific drug can also be resistant to other drugs with similar mechanisms of action. This condition is often seen in antibacterials with similar structures, such as the resistance observed between cephalosporins and penicillins. However, cross-resistance can sometimes occur between completely unrelated drug groups. An example of this is the cross-resistance between erythromycin and lincomycin (Jawetz et al., 1995; Mayer et al., 1995).

### Multi-drug resistance and pan-resistance

**FIGURE 1:**
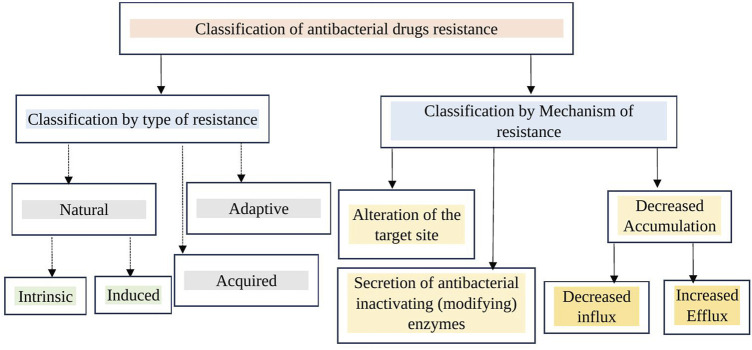
Classification of antibacterial drug resistance by type and mechanism. Antibacterial resistance can be categorized by type as natural, acquired, and adaptive. By mechanism, it includes target site alteration (modification), increased efflux (reducing intracellular concentration through efflux pumps), decreased entry of antibacterials into the bacterial cell (target site), and enzymatic inactivation.

Multidrug-resistant organisms are typically bacteria that no longer respond to the antibacterials designed to treat them, meaning these drugs can no longer effectively kill or control the bacteria. Multidrug resistance (MDR) in bacteria can develop through two main mechanisms. First, bacteria may acquire multiple genes, each providing resistance to a specific drug, often carried on resistance (R) plasmids. Second, MDR can arise from increased expression of genes responsible for multidrug efflux pumps, enzymatic inactivation, alterations in target structure, and other mechanisms. Bacterial strains resistant to three or more classes of antimicrobials are considered multidrug-resistant. If strains are resistant to all but one or two antibacterial groups, they are classified as extensively drug-resistant. Strains resistant to all available antibacterials are termed pan-drug-resistant (Eliopoulos et al., 2008; Nikaido, 2009; Manchanda et al., 2010). MDR in bacteria is a growing public health concern, characterized by the ability of pathogens to withstand the effects of multiple antibacterials. As a result, infections caused by multidrug-resistant bacteria are increasingly difficult to treat, necessitating urgent development of new antimicrobials and prudent use of existing antibacterials. Classification of antibacterial drugs resistance by type and mechanism of resistance has been depicted in the figure (Figure 1).

### Mechanisms of antibacterial drug resistance

With the recent increase in AMR, understanding the mechanisms by which bacteria resist antibacterials is becoming crucial for addressing this crisis (Zhu et al., 2013). To thrive in the presence of an antibacterial, bacterial strains must be able to interfere with several critical stages necessary for the antimicrobial drug to work effectively (Peterson and Kaur, 2018). There are four primary mechanisms by which bacteria develop resistance to antibacterials (Walsh, 2000). These include the enzymatic breakdown or structural modification of antibacterials, the use of efflux pumps to keep intracellular antibacterial concentrations below inhibitory levels, alterations to the antibacterial’s target site, and changes in the permeability of the cell membrane (Crofts et al., 2017). Bacterial attribute besides selection for antibacterial resistance genes (ARGs) that facilitates AMR is Biofilm formation (Davies et al., 2007; Canton and Morosini, 2011; Røder et al., 2016). The discussion of biofilm formation, wherein bacteria attach to surfaces and generate a shielding matrix, fostering a collective habitat that boosts their viability, has been addressed next to the discussion of the four primary resistance mechanisms.

Acquired resistance in bacteria commonly involves mechanisms like drug target modification, drug inactivation, and drug efflux. Conversely, intrinsic resistance mainly stems from limiting drug uptake, drug inactivation, and drug efflux. Gram-positive and Gram-negative bacteria exhibit structural differences, leading to variations in their drug resistance mechanisms. Gram-positive bacteria typically employ the restriction of drug uptake less frequently due to their lack of an outer membrane containing LPS and limited capacity for certain types of drug efflux mechanisms (Chancey et al., 2012; Reygaert, 2018). In contrast, Gram-negative bacteria have been observed to employ all four primary mechanisms of drug resistance (Abdus Salam et al., 2023). Therefore, the primary mechanisms of drug resistance, which include target alteration or modification, decreased accumulation, and enzymatic inactivation, have been elaborated in the next sections.

### Modification (alteration) of the drug target

One of the four primary mechanisms by which bacteria reduce the efficacy of antibacterials is by mutating, modifying, or shielding their cellular targets, thereby disrupting the binding of the antibacterials (Blair et al., 2015). Modifying or altering the antibacterial target of bacteria reduces or prevents the antibacterial’s ability to bind effectively, thereby diminishing its potency and effectiveness (Drlica and Zhao, 1997; Walsh, 2000; Wright, 2007; Munita and Arias, 2016; Peterson and Kaur, 2018). However, only those mutations that lead to reduced antibacterial binding without affecting the protein activity are favored (Smith et al., 2013). Bacteria can change the structure of their cell wall, membrane, or other cellular components that antibacterials target, making them less susceptible to these drugs. For example, some bacteria can alter their cell wall structure, reducing the binding sites for antibacterials that specifically target the cell wall, like beta-lactams (Schaenzer and Wright, 2020). Additionally, certain bacteria can undergo DNA mutations that change the structure of their ribosomes, which are the cell components responsible for producing proteins. These changes can hinder the ability of antibacterials, like macrolides and tetracyclines, to bind to the ribosomes and effectively inhibit bacterial growth (Wang et al., 2023). Hence, this form of resistance mechanism might arise from either mutations or enzymatic changes in the target site, or through target replacement or bypass. Each of these possibilities will be explained in detail below.

#### Target site alteration (by mutation or enzymatic alteration)

Bacteria have the ability to alter the targets necessary for drug binding, preventing the drug from binding effectively or at all to the modified target. These modifications often stem from spontaneous mutations in the gene or genes encoding the protein serving as the drug target. For example, mutations affecting the quinolone-resistance-determining region (QRDR) in DNA gyrase (topoisomerase II) and topoisomerase IV lead to the development of quinolone and fluoroquinolone resistance in both Gram-positive and Gram-negative bacteria (Drlica and Zhao, 1997; Aldred et al., 2014; Ashley et al., 2017). Another instance is seen in Rifampicin-resistant *Mycobacterium tuberculosis* strains, where mutations occur in the *rpoB* gene responsible for encoding the β-subunit of RNA polymerase, which is the target of rifampicin (Smith et al., 2013; Goldstein, 2014). Target site alteration happens when mutations in the molecules targeted by antibiotics stop the drugs from binding properly, making them ineffective. This mechanism poses a major challenge in treating infections as it can swiftly lead to the emergence of resistance in bacterial populations.

The most common mechanism of linezolid resistance involves mutations in genes encoding domain V of the 23S rRNA. Bacteria typically possess multiple copies of the 23S rRNA genes, and the number of mutated alleles correlates with an increase in minimum inhibitory concentration (MIC). Despite, clinically relevant resistance requires mutations in multiple alleles to occur. Additionally, mutations in the ribosomal proteins L3 and L4, which flank the linezolid binding site, have been linked to linezolid resistance (Miller et al., 2014). Resistance to linezolid, chloramphenicol, and clindamycin can also occur due to methylation of the 23S rRNA by an enzyme encoded by the *chloramphenicol–florfenicol resistance* (*cfr*) gene (Schwarz et al., 2004; Kehrenberg et al., 2005; Morales et al., 2010). Likewise, methylation of the *cfr* gene has been associated with the emergence of resistance in various bacteria, such as *Proteus vulgaris*, *Staphylococcus* spp., *Enterococcus* spp., *Bacillus* spp., and *E. coli* (Foster, 2017; Saha and Sarkar, 2021). It is notable that mutations in the 23S rRNA in Staphylococcal species are linked to decreased susceptibility to linezolid. Despite extensive clinical usage, leading to resistance selection in *S. aureus* and *Streptococcus pneumoniae*, linezolid remains effective in over 98% of staphylococcus species (Gu et al., 2015).

Furthermore, resistance to macrolide antibacterials is conferred by methylation of 23S ribosomal RNA (rRNA) through the action of *Erythromycin resistance methyltransferase* (*Erm*) (Blair et al., 2015). The *erm*(B) gene stands out as the predominant macrolide resistance determinant in *S. pneumoniae*. Induction of *erm*(B) facilitates substantial translation of *Erm*(B) when exposed to inducers like erythromycin (Chancey et al., 2011). The product of this gene dimethylates the target site of the 23S rRNA, which is A2058 in *E. coli* (Skinner et al., 1983; Weisblum, 1995a; Johnston et al., 1998). Methylation of the 23S rRNA by enzymes, encoded by various *erm* (*erythromycin ribosome methylase*) genes, leads to cross-resistance to macrolides, lincosamides, and streptogramin B. This phenomenon is termed the MLSB phenotype (Leclercq, 2002; Saha and Sarkar, 2021).

Bacterial reductases activate nitrofurantoin, leading to the formation of toxic intermediate compounds necessary for its antimicrobial activity. The primary mechanism of nitrofurantoin resistance involves mutations in the nitroreductase genes *nfsA* and *nfsB* (Whiteway et al., 1998; Osei, 2018). In addition, mutations in the *ribE* gene have been associated with nitrofurantoin resistance as well. The *ribE* gene encodes a lumazine synthase, an enzyme crucial for the biosynthesis of riboflavin, an essential cofactor for *nfsA* and *nfsB* (Osei, 2018).

#### Target replacement or target bypass

The development of β-lactam resistance in *S. pneumoniae* and methicillin resistance in *Staphylococcus aureus* is primarily attributed to the replacement of bacterial Penicillin-Binding Proteins (PBP). In *S. pneumoniae*, β-lactam resistance arises from the emergence of mosaic PBP genes. On the other hand, methicillin resistance in *S. aureus* results from acquiring the *mecA* gene, which integrates into the bacterial chromosomal DNA. The *mecA* gene, situated on a mobile genetic element, encodes Penicillin-Binding Protein 2a (PBP2a), a unique PBP with markedly reduced affinity for all β-lactams (excluding last-generation cephalosporins and carbapenems). Consequently, it enables uninterrupted cell wall synthesis even in the presence of β-lactams (Chambers, 1999; Moellering, 2012; Hiramatsu et al., 2013). *Staphylococcus* spp. demonstrates a considerable decrease in its susceptibility to β-lactam antibacterials, largely owing to an alternative penicillin-binding protein encoded by the *mecA* and *mecC* genes (Katayama et al., 2000; Pinho et al., 2001; Wendlandt et al., 2015; Foster, 2017). Target bypass or target replacement as an antibacterial resistance mechanism involves bacteria developing alternative pathways or substitute molecules that fulfill the same function as the original target, thereby evading the antibacterial’s effect. This strategy allows bacteria to survive despite the presence of antibacterials designed to inhibit essential cellular processes.

Resistance to glycopeptides in enterococci occurs through the acquisition of a group of genes known as van gene clusters. These genes facilitate the substitution of the glycopeptide target, specifically the terminal d-Alanine-d-Alanine moiety of peptidoglycan precursors, thereby decreasing the antibacterial molecule’s binding affinity. The alteration of the terminal d-Alanine-d-Alanine moiety to d-Alanine-d-Lactate results in high-level resistance, whereas the change to d-Alanine-d-Serine leads to low-level resistance (Miller et al., 2014). Instances of high-level vancomycin resistance in *S. aureus* (VRSA) have been reported, resulting from the acquisition of the *vanA* gene cluster from vancomycin-resistant enterococci (Sievert et al., 2008). Fortunately, this phenomenon continues to be rare (Christaki et al., 2020).

The acquisition of *dihydrofolate reductase* (*DHFR*) and *dihydropteroate synthase* (*DHPS*) genes, which encode trimethoprim-resistant DHFR enzymes and sulfonamide-resistant DHPS enzymes, respectively, has been documented as a cause of transferable resistance to these antimicrobial agents (Eliopoulos and Huovinen, 2001). The capacity of enterococci to utilize exogenous folinic acid might elevate the minimum inhibitory concentration (MIC) to trimethoprim-sulfamethoxazole *in vivo*, potentially leading to therapeutic failure when using trimethoprim-sulfamethoxazole to treat enterococcal infections (Zervos and Schaberg, 1985). In some cases, cells develop resistance by diverging from their typical physiological pathway, incorporating an alternative step. Typically, this involves the presence of an additional enzyme. For instance, in *E. coli* and *Citrobacter* sp., the production of an extra dihydrofolate reductase, determined by an R-plasmid, confers trimethoprim resistance. This additional enzyme differs from the chromosomal enzyme in its ability to bind to various anti-folate compounds (Pattishall et al., 1977). Additionally, resistance can occur through the massive overproduction of the antibacterial target, essentially overpowering the antibacterial. For instance, overproduction of DHFR has been documented as a cause of trimethoprim resistance in *E. coli* (Flensburg and Sköld, 1987; Eliopoulos and Huovinen, 2001).

#### Target site protection

Bacteria can produce a molecule that mimics the antibacterial target, binding to the antibacterial and decreasing its effective concentration. An example is the MfpA protein in *M. tuberculosis*, comprising pentapeptide repeats that mimic the shape and charge of B-DNA. MfpA provides resistance to quinolones by binding to DNA gyrase (the quinolone target) *in lieu* of DNA, thus reducing the availability of gyrase for quinolone binding (Hegde et al., 2005). In addition, Ribosomal protection proteins (RPPs) serve as an example of AMR through target site protection, and they have been identified in both Gram-positive and Gram-negative bacteria (Connell et al., 2003; Roberts, 2005).

### Decreased accumulation

Changing the cell wall or cell envelope permeability implies reducing entry or increasing the efflux of antibacterials, thereby regulating the internal concentration of antibacterials in the cell. Changes in pores can alter or inhibit the entering capability of antibacterials into the cell. Efflux can be increased specifically by acquisition of specific genes, as exemplified by tetracycline resistance (McMurry et al., 1980). Increased efflux can be due to the over-expression of physiologically present efflux pumps, causing in general a multidrug resistant phenotype (Pattishall et al., 1977). antibacterial entry into the cell is mainly through porins present in the outer membrane (Blair et al., 2015). Hence, decreased accumulation, which leads in decreased effectiveness of antibacterials, at the target site occurs due to a decreased cellular expression of porins or mutations in the porin genes leads to reduced entry of the antibacterial into the cell and possessing multidrug efflux pumps that are responsible for the active export of antibacterials from the cell.

### Decrease entry (influx) of antibacterials

Changes in the outer membrane permeability can impede the effective entry of antibacterials, resulting in decreased uptake by the bacterial cell (Walsh, 2000). Reducing the antibacterial’s ability to penetrate the microbial cell prevents it from reaching the antibacterial’s target within the bacteria (Peterson and Kaur, 2018). The absorption of antibacterials by Gram-negative bacteria is greatly influenced by porin channels. Mutations leading to the inactivation or downregulation of porin proteins, such as OprD, result in reduced permeability to various antibacterial classes, including aminoglycosides and fluoroquinolones (Reynolds et al., 2022). For antibacterials to be effective, they must first reach the target site and bind at concentrations sufficient to kill or inhibit bacterial growth. Therefore, any bacterial mechanism that obstructs the influx of antibacterials can lead to treatment failures.

LPS, a heavily acylated glycolipid, constitutes a major part of the outer membrane of Gram-negative bacteria, functioning as a permeability barrier for various chemicals, including antibacterials. This inherent resistance in Gram-negative bacteria reduces the permeability of certain antibacterials, thereby contributing to resistance (Nikaido, 1989; Blair et al., 2014; Choi and Lee, 2019; Salam et al., 2023). For instance, mycobacteria possess an outer membrane with a substantial lipid content, facilitating the penetration of hydrophobic drugs such as rifampicin and fluoroquinolones into the cell. However, this hydrophobic barrier limits the access of hydrophilic drugs (Kumar and Schweizer, 2005; Baran et al., 2023). Hence, to overcome the protective layer of the outer membrane of bacteria, bacteria utilize porins, which facilitate the passage of hydrophilic molecules of specific sizes (Fernández and Hancock, 2012).

Porins serve as the primary route of entry for hydrophilic antibacterials (such as β-lactams, fluoroquinolones, tetracyclines, and chloramphenicol) through the bacterial outer membrane. The quantity and variety of porins expressed on the outer membrane impact the penetration of hydrophilic antibacterials and consequently influence the susceptibility of the bacterial cell to these antibacterials (Fernández and Hancock, 2012; Choi and Lee, 2019). Additionally, acquired antibacterial resistance can arise from mutations that disrupt the expression of porins or their function (Nikaido, 1989; Ghai and Ghai, 2018). In a clinical strain of *Klebsiella pneumoniae*, for instance, a nonsense mutation in the OmpK36 porin gene prevents the correct translation of the protein (Wozniak et al., 2012). Mutations in porins can manifest in various ways, including porin loss, alterations in porin size or conductance, or reduced porin expression. Changes in porin expression typically result in low-level antibacterial resistance. However, mutations affecting porin expression can lead to high levels of resistance when combined with other co-existing mechanisms such as efflux pumps or enzymatic degradation of antibacterials (Fernández and Hancock, 2012; Ghai and Ghai, 2018).

## Increased expression of efflux pumps

Efflux pumps are indeed energy-dependent complex bacterial systems located on the cytoplasmic membrane. They have the capability to expel toxic molecules, including drugs, from the cell, thereby decreasing the concentration of the drug inside the cell and diminishing its effectiveness (Piddock, 2006; Soto, 2013; Munita and Arias, 2016; Abdi et al., 2020). Efflux pumps are widespread in various types of bacteria and can provide resistance to a wide array of antibacterials. They contribute significantly to multidrug resistance by expelling antibacterials from bacterial cells, reducing their intracellular concentration and efficacy (Abdi et al., 2020). The first efflux pump responsible for pumping tetracycline out of bacterial cells was identified in *E. coli* in 1980. It was encoded on a plasmid, which facilitated its spread among bacterial populations (Ball et al., 1980; McMurry et al., 1980; Nikaido and Pagès, 2012). When antibacterials reach the target site in adequate amounts, they can bind and produce the desired effect. However, mutations leading to the over-expression of efflux pumps can reduce the concentration of antibacterials at the target site, diminishing the drugs’ effectiveness.

Efflux pumps play a crucial role in microbial physiology by enabling microorganisms to regulate their internal environment. They achieve this by expelling various substances, including antimicrobial agents, metabolites, and quorum sensing signal molecules, thus maintaining cellular homeostasis and adaptation to environmental challenges (Pearson et al., 1999). The efflux pump mechanism aids the cell in removing antibacterial drugs, thereby contributing to antibacterial resistance in bacteria (Peterson and Kaur, 2018). Efflux pump-mediated antibacterial resistance is especially significant in bacteria like *Pseudomonas aeruginosa* and *Acinetobacter* spp. These pathogens commonly employ efflux pumps to expel antibacterials, contributing to their ability to withstand multiple antimicrobial agents (Walsh, 2000). Efflux systems typically have the ability to transport a wide range of unrelated substances, potentially leading to MDR (Piddock LJV., 2006; Piddock, 2006; Nikaido and Pagès, 2012).

Multidrug efflux mechanisms are predominantly encoded in the bacterial chromosome and can account for the inherent resistance of bacteria to certain antibacterials. Despite being broadly present across bacterial species, only a few of these efflux mechanisms contribute significantly to clinically relevant antibacterial resistance. Typically, clinical resistance arises from mutations that enhance pump expression or effectiveness (Piddock LJV., 2006; Piddock, 2006; Nikaido and Pagès, 2012). In contrast, genes encoding substrate-specific efflux pumps are often found on mobile genetic elements (Keith, 2005; Fernández and Hancock, 2012). Instances of substrate-specific efflux pumps include those tailored for tetracyclines, macrolides, and chloramphenicol (Keith, 2005; Poole, 2005; Reygaert, 2018).

Efflux pumps, which expel drugs and toxins from bacterial cells, are categorized into two types of bacterial transport proteins: primary and secondary transporters (Blair et al., 2014; Alenazy, 2022; Salam et al., 2023). Primary transporters belong to the ATP-binding cassette (ABC) family and are activated by ATP binding and hydrolysis to facilitate efflux. Secondary transporters, on the other hand, encompass various families, such as the major facilitator superfamily (MFS), resistance nodulation division (RND) family, small multidrug resistance (SMR) family, and multidrug and toxic compound extrusion (MATE) family (Poole, 2003; Blair et al., 2014; Reygaert, 2018; Hajiagha and Kafil, 2023; Palazzotti et al., 2023), and the drug metabolite transporter (DMT) superfamily (Blair et al., 2014). These secondary transporters utilize the energy generated by the electrochemical potential across the membrane to facilitate the efflux process (Gao et al., 2023).

Most of the efflux pumps found in Gram-positive bacteria are classified into the ABC and MFS families. These pumps are typically encoded by chromosomal genes, although some may also be carried on plasmids (Blair et al., 2014). The primary clinically relevant efflux systems in Gram-negative bacteria typically belong to the RND superfamily. These systems usually consist of an outer-membrane protein channel, a periplasmic protein, and a cytoplasmic membrane pump (Poole, 2003; Nikaido and Pages, 2012; Blair et al., 2014; Colclough et al., 2020). These efflux systems are capable of expelling a wide range of antibacterials as well as structurally unrelated molecules, including dyes and bile salts. Additionally, they can remove detergents and biocides commonly employed in medical settings (Nikaido and Pages, 2012). The upregulation of AdeABC, a multidrug RND efflux pump, is accountable for the drug resistance observed in *Acinetobacter baumannii* (Colclough et al., 2020). The augmented presence of RND efflux pumps renders *A. baumannii* resistant to a broad spectrum of antibacterials, including aminoglycosides. This resistance extends to fluoroquinolones, tetracycline, tigecycline, chloramphenicol, erythromycin, trimethoprim, netilmicin, and meropenem, reducing the susceptibility of the bacterium to these drugs (Ragueh et al., 2023). Quinolone resistance resulting from the overactivity of RND pumps has been extensively documented and is a prevalent occurrence (Lari et al., 2018). As a result, tigecycline is considered one of the last-resort antibacterials for treating infections caused by multidrug-resistant Gram-negative bacteria (Hornsey et al., 2011).

The tet system is one of the most extensively studied pumps driving efflux-mediated resistance (Beheshti et al., 2020). The tet system, belonging to the major facilitator superfamily, becomes activated only in the presence of tetracycline through tetR regulation. It expels tetracycline through a process of proton exchange, which provides the necessary energy. More than 20 tet genes have been identified, primarily located on plasmids, although they can also be present chromosomally, potentially carried on integrative conjugative elements (Grossman, 2016).

## Enhance secretion of antibacterial inactivating (modifying) enzymes

A clinically significant resistance mechanism is a bacterium’s capacity to produce enzymes that either break down or alter antibacterials, rendering them ineffective (Walsh, 2000; Egorov et al., 2018). The three main enzymes that deactivate antibacterials are β-lactamases, aminoglycoside-modifying enzymes, and chloramphenicol acetyltransferases (Kapoor et al., 2017; Peterson and Kaur, 2018; Reygaert, 2018). Drug resistance can occur when certain bacterial species inactivate antibacterials in one of two ways: either by degrading the antibacterial or by transferring a chemical group to it (Blair et al., 2015). In the structure of an antibacterial, hydroxyl and amide groups can be readily altered through hydrolysis (Mehta et al., 2016). Additionally, acetyl, phosphate, and nucleotide groups can be added to antibacterials, rendering them inactive (Mingeot-Leclercq and Decout, 2016). Once the antibacterial enters the cell, resistant bacteria either enzymatically degrade it or modify it to prevent it from binding to its target (Blair et al., 2015). For instance, the production of enzymes like β-lactamases, which break down the molecular structure of β-lactam antibacterials (such as penicillins and cephalosporins), rendering them inactive, is a key strategy used by pathogens (Benveniste and Davies, 1973; Walsh, 2000; Paraje and BrymanMéndez-Vilas, 2011; Egorov et al., 2018; Peterson and Kaur, 2018; Schaenzer and Wright, 2020). Additionally, chloramphenicol acetyltransferase acetylates chloramphenicol, while nine different enzymes can acetylate, phosphorylate, or adenylylate aminoglycoside antibacterials (Davies and Rownd, 1972; Benveniste and Davies, 1973). The enzymes secreted by Bacteria can inactivate or alter the structure of antibacterials, preventing the drugs from binding to their target and thus failing to kill or inhibit bacterial growth.

### β-lactamases

Genes encoding β-lactamases can be located in the chromosome or in mobile genetic elements (MGEs), which has facilitated their spread among bacteria. TEM-1, a plasmid-encoded β-lactamase, was first identified in Gram-negative bacteria in the 1960s. Since then, the introduction of new β-lactams has been followed by the emergence of new β-lactamases capable of degrading these compounds. For example, the introduction of third-generation cephalosporins in the early 1980s was quickly followed by the discovery of plasmid-encoded β-lactamases, known as Extended-Spectrum β-Lactamases (ESBLs), which can hydrolyze third-generation cephalosporins, in 1983 (Knothe et al., 1983).

β-lactamases are widely distributed and can be categorized into class A, which includes serine β-lactamases with active sites, and class B, consisting of metallo-β-lactamases that rely on a divalent metal ion, typically Zn^2+^, for their activity (Hall and Barlow, 2005), class C or AmpC β-lactamases and class D β-lactamases (Bo et al., 2024b).

A review by Salih and Ali explained Class A, C, and D β-lactamases function as enzymes through covalent ester intermediates, while class B relies on a zinc ion as a cofactor, making them metalloenzymes. Class A β-lactamases, such as ESBLs found in *E. coli* and *K. pneumoniae*, are notable. Class B β-lactamases include enzymes produced by various species like *Stenotrophomonas maltophilia*, *Bacteroides fragilis*, *Aeromonas*, and *Legionella*, capable of hydrolyzing carbapenems, penicillins, and cephalosporins. Class C β-lactamases, such as AmpC β-lactamases, are found in bacteria like *P. aeruginosa*, *Enterobacter cloacae*, *Citrobacter freundii*, and *Serratia marcescens*. Class D *β-lactamases* include oxacillin-degrading enzymes produced by Gram-positive cocci like *S. aureus*, which are induced by β-lactamases. Furthermore, classes of β-lactamases have been reviewed by (Salih Cesur Ali and Demiröz, 2013).

*β-lactamases* are capable of hydrolyzing a broad spectrum of β-lactam antibacterials that contain ester and amide bonds. This includes penicillins, cephalosporins, monobactams, and carbapenems (Livermore and Woodford, 2006; Diene et al., 2023). β-lactamases, hydrolyzing enzymes produced by members of the *Enterobacterales* family, are especially proficient at deactivating β-lactam antibacterials (Blair et al., 2015). The β-lactamases, initially referred to as penicillinases and cephalosporinases, deactivate the β-lactam ring structure by cleaving it at a specific site, rendering it incapable of binding to the target known as penicillin-binding proteins. Many members of the *Enterobacterales* family, as well as various species of Gram-positive bacteria like *S. aureus*, *Enterococcus faecalis*, and *Enterococcus faecium*, are recognized for carrying *β-lactamase* genes, which are transmitted through HGT (Blair et al., 2015).

ESBLs, a subset of class A β-lactamases, hold significant clinical relevance in Gram-negative bacteria. They confer resistance to penicillins, first-, second-, and third-generation cephalosporins, as well as aztreonam, but not cephamycins and carbapenems, which are inhibited by β-lactamase inhibitors. Additionally, ESBLs can also confer resistance to non-β-lactam agents such as fluoroquinolones, trimethoprim-sulfamethoxazole, nitrofurantoin, or aminoglycosides (Paterson and Bonomo, 2005; Wong and van Duin, 2017; Tamma et al., 2020). Furthermore, AmpC, a subclass of class C β-lactamase enzymes, provides resistance to penicillins, first-, second-, and third-generation cephalosporins, aztreonam, and cephamycins, but not carbapenems. Importantly, AmpC enzymes are not inhibited by β-lactamase inhibitors (Jacoby, 2009). Additionally, carbapenemases constitute a diverse group of enzymes that confer resistance to carbapenems, with many of them also providing resistance to almost all hydrolyzable β-lactams (Queenan and Bush, 2007).

### Enterobacteriaceae that produce ESBL and carbapenemase

Enterobacteriaceae comprise a large family of bacteria commonly responsible for infections both in healthcare settings and the community. Some strains of Enterobacteriaceae can produce extended-spectrum beta-lactamases (ESBLs), enzymes that degrade and render ineffective beta-lactam antibacterials. Carbapenems are among the few antibacterials effective against ESBL-producing bacteria, but resistance to them, facilitated by rising levels of resistance enzymes, is increasing. Certain Enterobacteriaceae can also produce carbapenemase enzymes, rendering carbapenems, penicillins, and cephalosporins ineffective. These bacteria, known as Carbapenem-Resistant Enterobacteriaceae (CRE), are often referred to as “nightmare bacteria” due to the limited availability of alternative antibacterials for treating their infections. Common Enterobacteriaceae species like *K. pneumoniae* (*K. pneumoniae*) and *Escherichia coli* can produce carbapenemase enzymes, including *K. pneumoniae* carbapenemase (KPC), Oxacillinase-48 (OXA-48), New Delhi Metallo-beta-lactamase (NDM), and Verona integron-encoded metallo-beta-lactamase (VIM) (CDC, 2019).

### Aminoglycoside inactivating enzymes

Aminoglycoside-modifying enzymes (AMEs) modify the structure of aminoglycoside molecules, decreasing their affinity and impeding their binding to the 30S ribosomal subunit. As a result, AMEs confer broad-spectrum resistance to aminoglycosides (AGs) and fluoroquinolones (FQs) (Strateva and Yordanov, 2009). AMEs have been detected in strains of *S. aureus*, *E. faecalis*, and *Streptococcus* pneumoniae (Kapoor et al., 2017; Ahmadian et al., 2021). AMEs facilitate aminoglycoside acetylation, phosphorylation, or adenylation, leading to modified antibacterials with reduced affinity for their target (Wright, 1999; Ramirez and Tolmasky, 2010; Petchiappan and Chatterji, 2017). The transfer of acetyl, phosphoryl, and adenyl groups is commonly observed for drug inactivation. Phosphorylation, adenylation, and acetylation are primarily utilized against aminoglycosides (Reygaert, 2018; Costa et al., 2020; Jannat et al., 2022).

### Acetylation

**FIGURE 2:**
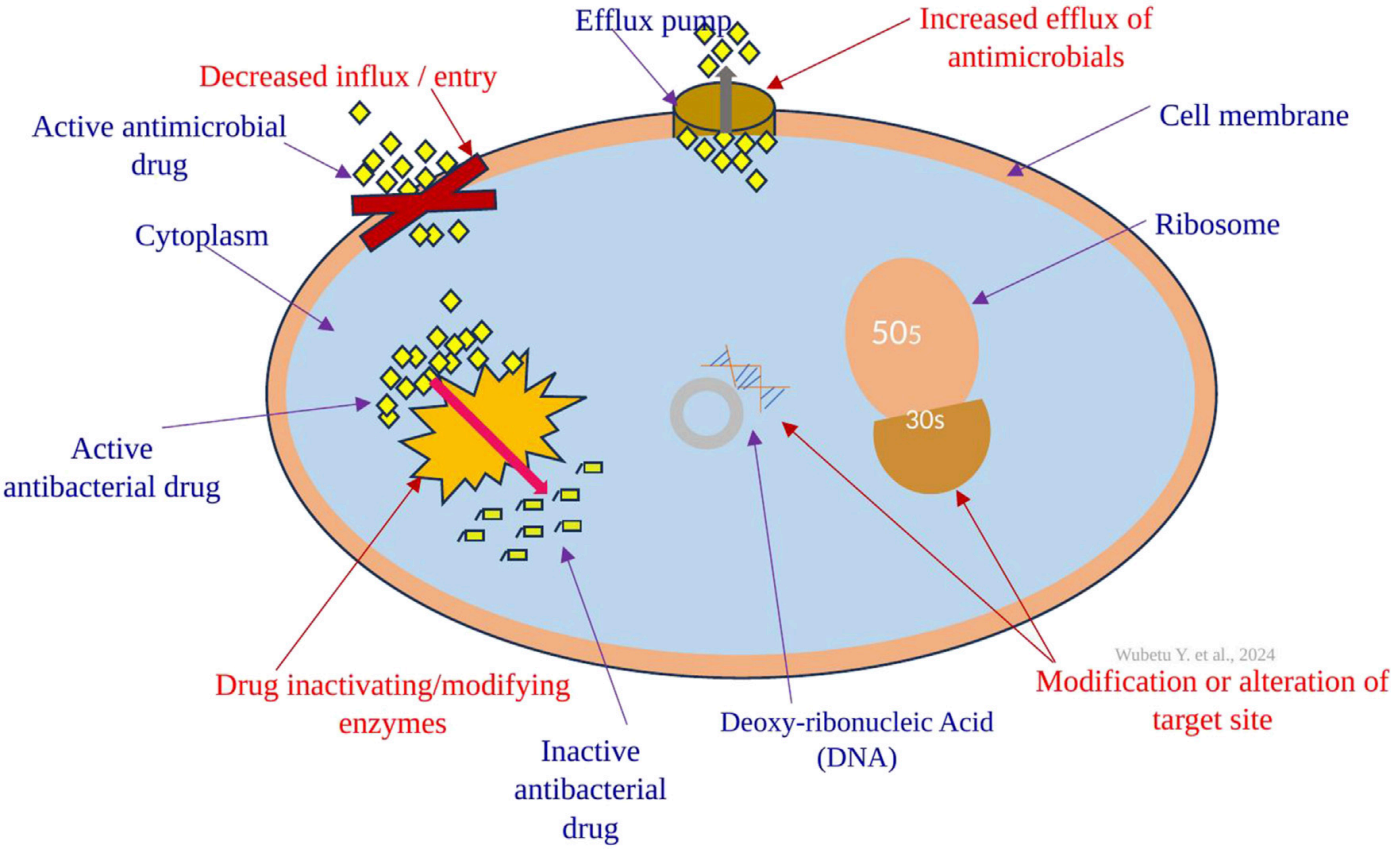
The primary mechanisms of antibacterial drug resistance include target modification (alteration) through, for example, ribosome alteration; restricting drug access using lipopolysaccharides (LPS) and porins; enhancing efflux via various efflux pumps (transporters); and modifying and inactivating the drug with different bacterial enzymes.

Other than aminoglycosides, acetylation is extensively utilized as a mechanism against chloramphenicol, streptogramins, and fluoroquinolones (Reygaert, 2018). For instance, resistance to chloramphenicol is observed in specific Gram-positive and Gram-negative bacteria, as well as certain strains of *Haemophilus influenzae*. These bacteria produce an enzyme known as chloramphenicol acetyltransferase, which acetylates the hydroxyl groups of chloramphenicol (Tolmasky, 2000). As a result of acetylation, this altered version of chloramphenicol loses its capability to properly bind to the 50S ribosomal subunit (Schwarz et al., 2004). Enzymatic acetylation of the antibacterial molecule stands out as the most common mechanism of chloramphenicol resistance. Numerous chloramphenicol acetyltransferases (CATs) have been identified across a broad spectrum of bacterial species (Schwarz et al., 2004). The primary mechanisms of resistance to antibacterial drugs have been demonstrated in the figure below (Figure 2).

### Antibacterial drug resistance involves more than one resistance mechanism

Resistance to the majority of antibacterial drugs involves more than one resistance mechanism. For instance, MRSA has evolved various drug-resistant mechanisms to ensure its survival. These include thickening of the cell wall, heightened activity of efflux pumps, mutation of drug targets, enzymatic modification of drugs, and the formation of biofilms (Singh et al., 2021). In addition, resistance to beta-lactam antibacterials can indeed be conferred through all the mentioned mechanisms, including inactivation by hydrolysis, enhanced efflux, reduced influx, and shielding of the antibacterial target (Kyriakidis et al., 2021).

Furthermore, quinolone resistance manifests through three distinct mechanisms: First, mutations in the target enzymes gyrase and topoisomerase IV; Second, resistance mediated by plasmids, facilitated by Qnr proteins, AMEs like AAC (6′)-Ib-cr and AAC (6′)-Ib-cr5, and plasmid-encoded efflux pumps; and third, resistance originating from the chromosome, arising from either decreased expression of porins or increased expression of chromosome-encoded efflux pumps (Su et al., 2005; Perez et al., 2007; Aldred et al., 2014). Moreover, resistance to aminoglycosides occurs based on several mechanisms: (1) enzymatic modification and inactivation of the aminoglycosides, and commonly observed across Gram-positive and -negative bacteria (Shaw et al., 1993; Ramirez and Tolmasky, 2010); (2) Resistance to aminoglycosides can also occur through increased efflux, reduced permeability, and modifications of the 30S ribosomal subunit, which disrupt the binding of the aminoglycosides (Cooksey et al., 1996). Moreover, examples of antibacterial drugs resistance with their mechanism of resistance have been shown in the table below (Table 1).

**TABLE 1:** Examples of antibacterial drugs resistance mechanisms.

Antibacterial drugs	Mechanism of resistance	References
Quinolone and fluoroquinolone resistance	mutations affecting the quinolone-resistance-determining region (QRDR) in DNA gyrase (topoisomerase II and topoisomerase IV)	[42,46,47]
Rifampicin-resistant *M. tuberculosis* strains	Mutations occur in the *rpoB* gene responsible for encoding the β-subunit of RNA polymerase	[43,48]
Linezolid, chloramphenicol, and clindamycin	methylation of the 23S rRNA by an enzyme encoded by the *cfr* gene	[49,50,51]
Linezolid resistance	mutations in genes encoding domain V of the 23S rRNA	[18]
Macrolides resistance	methylation of 23S ribosomal RNA (rRNA) through the action of Erythromycin resistance methyltransferase (Erm)	[2]
Nitrofurantoin resistance	mutations in the nitroreductase genes *nfsA* and *nfsB*	[60,61]
	mutations in the *ribE* gene	[60]
Methicillin resistance in *Staphylococcus aureus*	acquiring the mecA gene which encodes a different penicillin binding protein which is Penicillin-Binding Protein 2a (PBP2a)	[62-64]
Resistance to glycopeptides in enterococci	acquisition of a group of genes known as van gene clusters resulting in alteration of the terminal d-Alanine-d-Alanine moiety to d-Alanine-d-Lactate	[18]
Trimethoprim-sulfamethoxazole resistance in enterococci	The capacity of enterococci to utilize exogenous folinic acid	[70]
Trimethoprim resistance in *Escherichia coli*	overproduction of Dihydrofolate Reductase (DHFR)	[69, 72]
Aminoglycosides and fluoroquinolones Resistance	Mutations leading to the inactivation or downregulation of porin proteins, such as OprD, result in reduced permeability	[77]
Tetracycline resistance	Activation of the tet system (increased efflux)	[107], [108]
β-lactam antibacterials resistance	production of β-lactamases	[32,33,44,109,113,114]
Chloramphenicol resistance	Acetylation by chloramphenicol acetyltransferase	[114,115]
Aminoglycoside resistance	Modification of the structure of aminoglycosides by Aminoglycoside-modifying enzymes (AMEs)	[127]

### Biofilm formation

A biofilm is a stationary, three-dimensional matrix composed of microscopic organisms that have clustered together on a surface to establish a colony (Sharma et al., 2019). Bacterial biofilms are communities of bacteria enclosed within an extracellular polymeric substance (EPS) (Zhao et al., 2023). Biofilms are microbial communities that adhere to either biotic or abiotic surfaces, where the cells are encased in a self-produced matrix. Medically, biofilms are significant as they have been implicated in the pathogenesis of various bacterial infections that are challenging to eradicate with antibacterials. They achieve this by reducing the penetration of the drug into bacterial cells and by acting as a physical barrier that prevents the drug from reaching its target (Hoyle and Costerton, 1991; Costerton et al., 1999; Van Acker et al., 2014; Gebreyohannes et al., 2019; Dutt et al., 2022). *Pseudomonas aeruginosa* biofilms are known to cause chronic lung infections in patients with cystic fibrosis (CF) (Singh et al., 2000). *Staphylococcus aureus* biofilms have the capability to inhabit implanted medical devices like pacemakers (Marrie et al., 1982). Beyond the four antibacterial resistance mechanisms, biofilm formation by bacteria further complicates infection management. Biofilms prevent antibacterials from reaching and binding to the target sites within each bacterium due to the protective EPS, making such infections difficult to treat.

As previously noted, biofilms are generated as a means of survival, serving either to facilitate infection establishment or to enable survival on non-living surfaces, thereby aiding in their transmission (Zhao et al., 2023). Biofilms are notably intricate because they consist of both the EPS and a metabolically dormant “core.” This core effectively avoids detection by the immune system through sequestration and withstands antibacterial assault by reducing its metabolism, thereby diminishing the efficacy of antibacterials. Additionally, the structural EPS barrier restricts the infiltration of immune cells and antibacterials (Yin et al., 2019). Biofilms serve not only as virulence factors that facilitate infection establishment or transmission but also as crucial mechanisms for phenotypic resistance. This dual role underscores their significance, as they are frequently implicated as key drivers of infection recurrence or resistance to treatment, posing a significant concern in clinical settings (Gilbert and McBain, 2001).

The resistance of infectious biofilms to antibacterial treatment primarily stems from two main factors: poor penetration of antibacterials into biofilms and intrinsic antibacterial resistance. Firstly, antibacterial-susceptible bacteria are typically eradicated when in a suspended or planktonic state, but within biofilms, only surface bacteria are reached and killed, while those deeper within remain unaffected. In contrast, antibacterial-resistant bacteria are neither eliminated in a planktonic state nor at the biofilm’s surface. Secondly, poor antimicrobial penetration into the depths of biofilms is a common issue. This is due to reduced diffusion of antimicrobials and their adsorption onto the protective matrix of EPS produced by the biofilm. Nutrients and metabolic waste products are transported through water channels within the EPS matrix. It is worth noting that within biofilms, the pH (approximately 5–9) tends to be lower than the physiological pH found outside of infectious biofilms (Davies, 2003; Koo et al., 2017).

Numerous mechanisms are reportedly accountable for AMR in biofilm structures. The first reason is there is limited diffusion of antibacterials through the biofilm polysaccharide matrix, although certain antibacterials may still manage to penetrate it (Anderl et al., 2000). The other mechanism is physiological changes resulting from slow growth rates and responses to starvation, including oxygen and nutrient deprivation, or environmental stress (Brown et al., 1988; Walters et al., 2003). The third mechanism is due to phenotypic changes in the cells composing the biofilm. The other is because of quorum sensing, although its precise role is not yet fully understood (Brooun et al., 2000). The fifth reason is due to the expression of efflux pumps (Gilbert et al., 2007). The last mechanism responsible for AMR in biofilm is related to Persister cells, which are small fractions of bacteria that exhibit persistence by resisting elimination when exposed to antimicrobials. Notably, these persistent cells are not mutants (Gilbert et al., 2002).

The organisms secrete adhesive proteins and extracellular matrix, which serve to anchor the cells to a surface and shield the colony from dislodgement, environmental threats, host immune responses, and antimicrobial agents (Jacqueline and Caillon, 2014). Moreover, individual bacteria within biofilms, which have been subjected to elevated concentrations of antibacterials, can endure and subsequently reestablish a more resilient biofilm. This phenomenon is recognized as recalcitrance (Ciofu et al., 2022). As a result, biofilms often exhibit resistance to antibacterial treatment and may consequently necessitate surgical intervention. Nevertheless, surgical measures may not always succeed, leading to substantial morbidity and mortality. Biofilms are implicated in over 500,000 deaths annually in the United States alone (Charani and Holmes, 2019).

Antibacterials that manage to penetrate the deep layers of a biofilm may exhibit reduced effectiveness due to conditions resulting from bacterial metabolism and diffusion limitations. These processes give rise to gradients in oxygen, cations, and other solutes, as well as fluctuations in pH, all of which can influence the uptake and efficacy of antibacterials (Stewart and Franklin, 2008; Thomas et al., 2012). Furthermore, the slow or halted growth of cells deep within the biofilm is recognized to diminish antibacterial susceptibility in biofilms (Wood et al., 2013). Metabolic responses to nutrient limitation may govern antibacterial tolerance in growth-arrested cells under these circumstances (Nguyen et al., 2011; Amato et al., 2014). Bacterial populations generate persister cells, which remain in a dormant state and are unaffected by antibacterials. These cells play a significant role in the high tolerance of biofilms to antibacterials. (Keren et al., 2004). Metabolic cooperation within biofilms can also contribute to antibacterial resistance, a phenomenon observed particularly in polymicrobial biofilms (Dalton et al., 2011). This resistance is likely attributed, at least in part, to enhanced productivity and increased thickness of the matrix within these assemblies (Brenner and Arnold, 2011). Diversity within biofilms may suggest a form of division of labor (Estrela and Brown, 2013). where mutualistic interactions between taxa result in overall increased productivity (Vega and Gore, 2014).

It could be argued that the primary drivers of AMR are not the classical drug resistance mechanisms, such as efflux pumps, target site modification, or enzymatic degradation. Instead, it is likely that the matrix of biofilms serves as a mechanical and biochemical shield, creating conditions that attenuate the activity of drugs, such as low oxygen levels, low pH, high carbon dioxide levels, and limited water availability. Under these circumstances, conventional antibacterials struggle to eliminate bacteria effectively. Furthermore, when bacteria experience nutrient scarcity, they may become tolerant to antibacterials. This phenomenon may account for the apparent higher antibacterial resistance of cells within the deep layers of a biofilm. Notably, bacteria extracted from biofilms and cultured in broth regain their full susceptibility to antibacterials, indicating that the resistance observed is phenotypic rather than genotypic (del Pozo and Patel, 2007).

Biofilms are implicated in approximately 80% of human infections (Yasir et al., 2020). Biofilm formation is one of the key factors contributing to the development of tolerance against antimicrobial agents (Flemming et al., 2016). Between 50% and 70% of nosocomial infections are caused by biofilm formation on implanted medical devices (Cangui-Panchi et al., 2022) *S. aureus* biofilms have the ability to shield cells from adverse conditions, such as nutrient limitation, extreme temperatures, dehydration, and even antibacterial drugs (Lee et al., 2020; Idrees et al., 2021; Guo et al., 2022; Cangui-Panchi et al., 2023). The formation of biofilms by certain bacteria, including *E. faecalis*, *S. aureus*, *Staphylococcus epidermidis*, *Streptococcus viridans*, *E. coli*, *K. pneumoniae*, *Proteus mirabilis*, *and P. aeruginosa*, represents another mechanism of AMR exhibited by these pathogens (Van Acker et al., 2014; Dutt et al., 2022). In healthcare settings, the most common pathogens found in biofilms include *S. aureus*, *P. aeruginosa*, *A. baumannii*, *and K. pneumoniae* (Høiby et al., 2010).

One of the primary challenges in using antibacterials to treat biofilms is reaching the necessary MIC of the drug at the site of infection. The MIC for a biofilm can be between 10-1000 times greater than the MIC for planktonic cells (Jacqueline and Caillon, 2014; Paharik Alexandra and Horswill Alexander, 2016). Due to biofilms are up to 1000 times more resistant to antibacterials compared to their planktonic or free-floating counterparts (Potera, 2010), even after treatment, the host may retain subpopulations of bacteria, allowing for the potential reestablishment of infection (Leanse et al., 2023).

Biofilm formation occurs naturally through quorum sensing, which involves the detection of extracellular autoinducers. The higher cell density within a biofilm enhances its resistance to antibacterials compared to planktonic cells. This increased resistance is attributed to the reduced ability of antibacterials to diffuse through the biofilm matrix, as well as to quorum sensing and the expression of efflux pumps. Efflux pumps, recognized as one of the main contributors to resistance, enhance antibacterial tolerance in biofilms by blocking membrane channels, altering the chemical structure of antibacterials, and preventing the action of multidrug pumps (Zhang and Mah, 2008). Quorum sensing (QS), also known as cell-to-cell signaling, refers to the regulated expression of specific genes in response to extracellular chemical signals produced by bacteria themselves (Pearson et al., 1999). Quorum sensing is a bacterial communication process used to collectively regulate group behaviors (Ng and Bassler, 2009; Rutherford and Bassler, 2012). It is widely recognized that QS plays a crucial role in the development of biofilms. The relationship between QS and biofilm formation is often referred to as sociomicrobiology (Parsek and Greenberg, 2005). By inhibiting this communication between cells, there may be a potential avenue to prevent the spread of AMR (Brackman et al., 2016). The role of quorum sensing on biofilm formation has been reviewed by Clayton W. Hall and Thien-Fah Mah (Hall and Mah, 2017).

The reduced penetration of antibacterial drugs into biofilms enables MRSA to survive even in the presence of drugs at lower concentrations (Idrees et al., 2021). Therefore, MRSA biofilm-associated infections are complex and challenging to eradicate (Idrees et al., 2021). In addition to the extensive secretion of virulence factors, biofilm formation is a significant feature that protects *S. aureus* from host defense mechanisms and eradication measures (Olsen, 2015; Paharik Alexandra and Horswill Alexander, 2016; Cangui-Panchi et al., 2022). *Acinetobacter baumannii* has the ability to form biofilms, thereby prolonging its survival on medical devices such as ventilators in intensive care units (ICUs) (Pakharukova et al., 2018).

#### Horizontal gene transfer (HGT)

Resistance traits can be passed down from one generation to another through inheritance. Additionally, AMR genes can be acquired through HGT between bacteria, facilitated by mechanisms such as conjugation, transformation, or transduction (CDC, 2019; Collineau et al., 2019; Mellor et al., 2019; Shehreen et al., 2019; Tsigalou et al., 2020; De Lucia et al., 2021). Resistance genes have the capability to provide resistance to a particular antibacterial or a group of antibacterials, and they can be situated on plasmids, transposons, or other mobile genetic elements that have the ability to transfer between bacterial cells (Shehreen et al., 2019). HGT can take place in various environments, including soil, water, the digestive systems of humans and animals, and within food sources. The transfer of AMR genes, along with their persistence in bacterial populations and the development of multidrug resistance, is significantly facilitated by genetic elements such as plasmids, integrons, and transposons (Aarestrup, 2006; Salyers et al., 2007; Bennett, 2008; Revilla et al., 2008). Generally, antibacterial resistance genes can be inherited or acquired via HGT.

**FIGURE 3:**
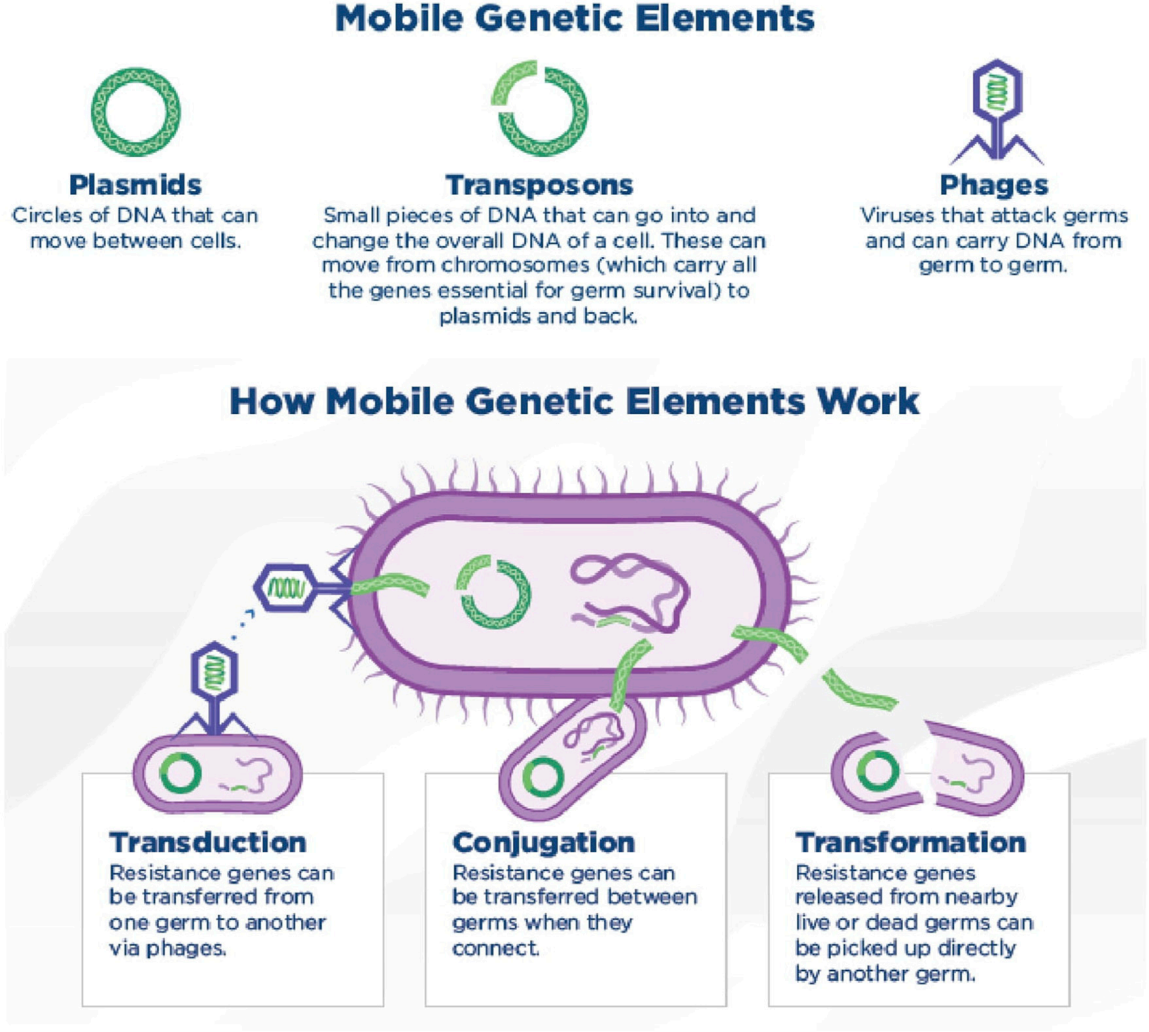
Mobile genetic elements and how they transmit via horizontal gene transfer methods. Conjugation (via the direct transfer of DNA between two bacterial cells through cell-to-cell contact.), transduction (transfer of genetic material from one bacterium to another by a bacteriophage) and transformation (uptake of free DNA from the environment by a bacterial cell) are the three mechanisms of horizontal gene transfer methods. (CDC, 2019). (Copyright: CDC, 2019 CC BY 4.0, available at http://dx.doi.org/10.15620/cdc:82532)

Microbial species maintain resistance to antibacterials not only by transferring resistance genes down to their offspring but also by possessing the capability to transfer genes horizontally between different species, a process known as HGT (Touchon et al., 2017). Vonwintersdorff *et al.* recently reviewed HGT more thoroughly (von et al., 2016) Mobile genetic elements and how they transmit via horizontal gene transfer methods has been shown (Figure 3).

##### Conjugation

Conjugation stands as one of the most impactful mechanisms of HGT. This process necessitates direct physical interaction between bacterial cells, enabling the transfer of genetic material. During conjugation, a sex pilus is formed, facilitating the transfer of a plasmid to the recipient bacterium. In a single conjugation event, multiple drug resistance genes located on the plasmid can pass to the recipient bacterium, resulting in the acquisition of multidrug resistance (Graf et al., 2019; Wang et al., 2019; Gonzalez-Villarreal et al., 2022).

Conjugation involves the transfer of DNA between live bacterial cells and necessitates direct contact between the donor and recipient cell. AMR genes are frequently found on mobile genetic elements (MGEs) like plasmids and transposons, often associated with insertion elements, integrons, and genomic islands. Transposons and insertion sequences can move within bacterial cells, while plasmids and other mobile genetic elements can lead to complex arrangements. Conjugation can encompass the transfer of conjugative or non-conjugative plasmids or transposons, with the latter now classified as Integrative Conjugative Elements (ICE) (Burrus et al., 2002; Burrus and Waldor, 2004; Wozniak and Waldor, 2010) or Integrative Mobilizable Elements (IME) (Lyras et al., 2004).

ICEs and IMEs also include genomic islands. The transfer of a non-conjugative plasmid can occur when other mobile elements provide the essential transfer genes (tra genes). These transfer genes, also known as tra genes or transfer operons, are crucial for the non-sexual transfer of genetic material between both Gram-positive and Gram-negative bacteria. Plasmid fusion is often enabled by the presence of insertion elements from transposons on the plasmids. Mobile genetic elements contribute to genomic plasticity, though they often exhibit plasticity themselves (Doublet et al., 2005; Brochet et al., 2008). An example of plasticity is the IME known as *Salmonella* genomic island 1 (*SGI1*), which is found in various *Salmonella* serovars and in *P. mirabilis.* The originally discovered SGI1 contained resistance genes for ampicillin, chloramphenicol, florfenicol, streptomycin, spectinomycin, sulfonamides, and tetracycline. In fact, many variants of *SGI1* have been identified (Doublet et al., 2005; Douard et al., 2010; Hall, 2010). It has even been shown that during a 10-year period, in a same *Salmonella Agona* clone, only the SGI1 changes while the genetic background of the strain remains the same (Doublet et al., 2004).

However, conjugation is constrained by several molecular and epidemiological factors. First, the ecosystem must facilitate contact between the strains. Additionally, the strains must have some degree of mobility, either independently or through external influences. Third, plasmid incompatibility can prevent the transfer of plasmids belonging to the same incompatibility group into the same cell. Fourth, particularly for IMEs, the genetic background of the cell must support the integration of the IME. Lastly, some mobile genetic elements possess maintenance systems that can cause cell death if these elements are expelled from the cell. Bacteria have developed various systems for plasmid transfer, but certain fundamental conjugative steps are common to all these systems. In Gram-negative bacteria, conjugation generally follows a mechanism that begins with the formation of conjugative pili to facilitate contact between donor and recipient cells. In Gram-positive bacteria, different mechanisms are used to establish cell contact, such as pheromone-induced plasmid transfer in enterococci (Palmer et al., 2010). or aggregation-mediated plasmid transfer in *Bacillus thuringiensis* subsp. *israelensis* (Andrup et al., 1993).

A crucial characteristic of plasmids is their host range, as this dictates how extensively they can disseminate antibiotic resistance or other traits without physically recombining with the DNA of a new host. HGT can only impact bacteria that are proficient in gene exchange. Comparisons of bacterial genomes have shown that members of “exchange communities” tend to be similar in factors such as genome size, genome G/C composition, carbon utilization, and oxygen tolerance. (Jain et al., 2003). Host range generally seems to be restricted by the interaction between the plasmid and its gene products with the host’s enzymatic machinery. Host range is not an all-or-nothing trait; rather, in the environment, certain species or strains are favored among potential hosts. (Heuer and Smalla, 2007), on the other hand, MGEs are typically not fixed globally but persist in patches of local subpopulations (Berg and Kurland, 2002). A detailed explanation on mechanism of conjugation has been reviewed by Claire Verraes, *et al.* (Verraes et al., 2013)

##### Transformation

Transformation is a process of DNA recombination wherein exogenous DNA fragments (naked DNA) from the environment are taken up by a microorganism and become integrated into its genetic makeup, becoming inheritable traits (Kelly et al., 2008; Winter et al., 2021). Transformation involves several steps. Initially, bacterial DNA is released from bacterial cells, either passively following death and lysis or actively at specific points in the growth cycle for certain bacteria (Lorenz and Wackernagel, 1994; Matsui et al., 2003). Following release, the DNA is taken up by competent bacteria nearby. Subsequently, the DNA must withstand the potentially destructive action of nucleases within the bacterial cell and become stably incorporated into the acceptor cell. Finally, the incorporated DNA is expressed. In theory, any bacterial chromosomal or extra-chromosomal DNA can be transferred via transformation. Some bacterial species are naturally competent for this process, such as *Campylobacter* spp., *Bacillus subtilis*, and particularly *Streptococcus* species (Seitz and Blokesch, 2013).

Only for a rather limited number of bacterial species have the natural transformation systems been studied in great detail (Dubnau, 1999): *B. subtilis*, *S. pneumoniae*, *H. influenzae*, *Neisseria gonorrhoeae*, *Acinetobacter* sp., *Pseudomonas stutzeri*, *Helicobacter pylori*. In Gram-positive model bacteria studied, the initial step in transformation involves the binding of double-stranded DNA to the cell, without preference for specific base sequences. Subsequently, the bound DNA is fragmented, with the transported single strands passing across the membrane while the non-transported strand is degraded. In contrast, efficient DNA uptake in Gram-negative model organisms like *H. influenzae* and *N. gonorrhoeae* requires the presence of specific uptake sequences typically located in the inverted repeats of donor sequences. Once bound, the DNA quickly becomes resistant to DNase and is taken up after fragmentation. Beyond differences in DNA uptake processes, bacteria also vary in their efficiency of integrating incoming DNA through heterologous recombination. ((Lorenz and Wackernagel, 1994; Sikorski et al., 2002).

The induction of competence varies depending on the species. Some naturally transformable species, like *Acinetobacter* spp., remain competent throughout the logarithmic growth phase. Others, such as *S. pneumoniae*, are competent for only brief periods. For example, *B. subtilis* develops competence only at the onset of the stationary phase. On the contrary, competence can also be expressed constitutively, as observed in *N. gonorrhoeae* (Johnsborg et al., 2007). Differences in the cell wall structure between Gram-positive and Gram-negative bacteria result in variations in their DNA uptake systems, despite their utilization of similar proteins (Chen et al., 2005). In certain bacterial species like *E. coli*, competence can be induced *in vitro* through chemical or physical means such as the presence of CaCl_2_, EDTA, temperature shifts, electroshocks, or exposure to light (Davison, 1999; Cérémonie et al., 2004; Cérémonie et al., 2006).

The prerequisites for transformation include the availability of free DNA, the development of competence, and the uptake and stable integration or autonomous replication of the acquired DNA. However, there is limited understanding of the significance of natural transformation in various environmental settings for bacterial adaptability. Transformation could play a crucial role in the establishment, maintenance, and gene transfer within bacterial biofilms. (Molin and Tolker-Nielsen, 2003; Petersen et al., 2005). Reports have indicated that despite the presence of ubiquitous DNases, high molecular weight free DNA can be detected in various environments. It is hypothesized that free DNA released from microorganisms or decaying plant material can serve as a nutrient source or as a reservoir of genetic information for indigenous bacteria. There have been published reports on the persistence of nucleic acids in non-sterile soil (Nielsen et al., 1997a; Blum et al., 1997). In addition, microbial activity has been identified as a significant biotic factor influencing the persistence of free DNA in soil. Increased microbial activity often correlates with heightened DNase activity in the soil. (Blum et al., 1997). Cell lysates from *Pseudomonas fluorescens*, *Burkholderia cepacia*, and *Acinetobacter* spp. were used as a source of transforming DNA for *Acinetobacter* sp. populations in both sterile and non-sterile soil environments for a brief period of a few days. Nielsen *et al.* demonstrated that cell debris can shield DNA from degradation in soil. Cell walls likely play a crucial role in safeguarding DNA after cell death. (Nielsen et al., 1997a; Paget and Simonet, 1997).

##### Transduction

Transduction is a process of bacteriophage-mediated transfer, wherein genetic material is exchanged between a bacteriophage and an infected bacterium through the action of the bacteriophage, which is a virus that infects bacteria. Unlike transformation, which involves the uptake of naked DNA from the environment, transduction relies on the transfer of DNA via bacteriophages (Reygaert, 2018). Initially, the bacteriophage attaches to the bacterium and injects its genetic material, which may include both viral and host bacterial DNA. Once inside the bacterial cell, the foreign DNA needs to be stabilized, either by forming an autonomously replicating element or by integrating into the bacterial DNA. Once stabilized, the foreign DNA can direct the production of new phage particles. Through this process, bacterial plasmid and/or genomic DNA of various lengths can be transferred from one bacterium to another, depending on the specific bacteriophage involved. Transduction typically occurs between closely related bacterial strains due to the host specificity of bacteriophages. However, the transducing capacity of a phage is not necessarily limited to bacteria it can infect and may extend to a wider range of bacterial species (Jensen et al., 1998; Holmfeldt et al., 2007). Hence, transduction is a vector (bacteriophage)-mediated transfer of drug resistant gene from resistant bacteria to a non-resistant bacterium.

So far, the transfer of AMR genes via transduction has been infrequently reported. However, in the case of *S. aureus*, instances of the transfer of the plasmid-borne *qacB* gene, which encodes a multidrug efflux protein, and the transfer of AMR plasmids via transduction have been documented (Nakaminami et al., 2007; Varga et al., 2012). In addition to mobile genetic elements, integrons—a type of transposon—can transfer to other bacteria, facilitating bacterial evolution by acquiring new genes. While DNA can theoretically be randomly inserted into a nonhomologous end of a DNA sequence, the probability and frequency of this event are exceedingly low. Natural transformation can also lead to the integration of AMR genes into the bacterial genome. (Jiang et al., 2017).

Many pathogenicity determinants, such as toxins, have been acquired via phages. Examples include Corynebacterium diphtheriae, *Clostridium botulinum*, *Streptococcus* pyogenes, *S. aureus*, and Shiga toxin-producing *E. coli* (*E. coli*). (Brüssow et al., 2004). Although most bacteriophages infect only a narrow range of hosts, this mechanism of gene transfer has the advantage that transducing phages can be rather persistent under environmental conditions, do not require cell-cell contact, and DNA in transducing phage particles is protected (Wommack and Colwell, 2000). Furthermore, marine environments are probably a major setting for virus mediated gene transfer between bacteria, where there is an estimated abundance of greater than 10^29^ virus particles (Hendrix et al., 1999; Weinbauer and Rassoulzadegan, 2004).

### Measures (interventions) implemented in tackling AMR

Despite the acknowledgment of antimicrobial resistance as a global crisis needing immediate attention, there has been minimal advancement in raising awareness about antimicrobial resistance, tracking antimicrobial consumption, executing infection prevention and control programs, and enhancing the use of antimicrobials in the human sector (Global Database for Tracking Antimicrobial, 2023). To tackle this threat effectively, ongoing and vigorous measures are necessary, such as preventing infections from occurring initially, reducing the development of resistance through better antibiotic usage, and halting the spread of resistance when it emerges (CDC, 2019). The emergence of Antimicrobial Stewardship Programs (AMS) as a strategy aims to combat the spread of antimicrobial resistance, enhance patient clinical outcomes, and manage costs effectively (Montrucchio et al., 2019). Moreover, AMS is crucial in healthcare settings as it encourages the prudent use of medications and reduces toxicity and cost.

In 2015, the WHO initiated a Global Action Plan on AMR to tackle a challenge described by its director general as: “threatening the very core of modern medicine and the sustainability of an effective global public health response to the enduring threat of infectious diseases.” (World Health Organization, 2015) The WHO and its Global Action Plan broadly outline five strategic objectives to combat AMR: (1) enhance awareness and understanding of AMR; (2) strengthen knowledge through surveillance and research to combat infections with control measures; (3) implement effective sanitation, hygiene, and infection prevention strategies; (4) optimize the use of antimicrobials in both human and animal health; and (5) promote sustainable investment in new medicines, diagnostic tools, and vaccines (WHO, 2015). Additionally, the development and availability of faster diagnostic tools and accurate antimicrobial profiling for targeted antibiotic therapy are crucial (WHO, 2015).

The WHO reported high levels of bacterial AMR globally, emphasizing the necessity of a One Health approach to address the AMR crisis. This approach operates at local, national, and global levels, involving collaboration among policymakers, stakeholders, practitioners, and researchers (Zhuo et al., 2018; Mouiche et al., 2019). One Health is an integrated, unifying approach designed to sustainably balance and optimize the health of people, animals, and ecosystems (One Health High-Level Expert Panel et al., 2022). One Health recognizes that the health of humans, domestic and wild animals, plants, and the wider environment (including ecosystems) are closely linked and interdependent. Therefore, addressing global health issues necessitates a multisectoral, multidisciplinary response to AMR at this One Health interface (World Health Organization, 2023).

WHO, the United Nations (UN), and the European Union (EU) have made efforts to reduce and restrict antimicrobial use in animals. This includes legislating bans on the use of certain antibiotics in agrifood systems for growth promotion and promoting antimicrobial stewardship in treating food animals and small domestic pets. However, implementing these controls can be challenging, especially in developing countries where the demand for food animals continues to increase annually (Pokharel et al., 2020). Additionally, WHO has developed the Global Action Plan for managing antimicrobial resistance (GAP-AMR) and subsequently launched the Global Antimicrobial Resistance and Use Surveillance System (GLASS). These initiatives aim to continuously address existing knowledge gaps and work towards achieving the goals of the GAP-AMR program (World Health Organization, 2021). Among the newly proposed measures, one of the most effective approaches is to enhance public awareness of the AMR pandemic as a preventive strategy. This requires effective communication with all stakeholders (Mostafa et al., 2021).

To contain and control AMR, coordinated efforts and collaboration are required within and between various sectors, including healthcare industries, pharmacy, agriculture, finance, trade, education, and nongovernmental organizations, at both national and international levels (Control). To combat AMR, key focuses include rational antibiotic prescribing, restricted use of prophylactic antimicrobials, patient education, adherence to antibiotic therapy, and maintaining appropriate hospital hygiene through antimicrobial stewardship (Majumder et al., 2020).

To support the development of tools for antimicrobial stewardship programs at local, national, and global levels, the Expert Committee classified antibiotics into three groups: “Access,” “Watch,” and “Reserve.” This classification aims to improve access and clinical outcomes, reduce the risk of antimicrobial resistance, and preserve the use of “last-resort” antibiotics for those who need them (Mudenda et al., 2023). The Access group consists of first- or second-line empiric therapies for many common conditions. These antibiotics typically have a narrower spectrum and a low risk of toxicity, and they should be readily available in all hospitals (Leekha et al., 2011). The Watch group includes antibiotics that have a higher risk of toxicity or a greater potential to induce resistance. Antimicrobial stewardship programs should restrict their use to only recommended indications (Aricò et al., 2023). In managing severe or life-threatening infections caused by multidrug-resistant bacteria, the Reserved category of drugs is used as a last-resort option. These drugs should be protected from inappropriate use through strict restrictions and approval programs (Ranjalkar and Chandy, 2019). This classification was introduced by the WHO in 2017 and is updated every 2 years. The WHO updated its classification in 2023, setting a country-level target that at least 60% of total antibiotic consumption should consist of Access group antibiotics (AWaRe classification of antibiotics for, 2023).

#### Strengthening rational antimicrobial use

“The WHO defines "rational use of medicine" as using the correct medications, including antibiotics, that are appropriate for the clinical needs of patients. This involves administering exact doses tailored to individual needs, for an adequate duration, and at the lowest cost (Lin et al., 2022). Another strategy to address antibiotic resistance is by reducing antibiotic usage. This can be achieved through educational initiatives and guidelines for healthcare workers. Additionally, ongoing surveillance is essential to detect resistance in new strains and monitor increases in resistance rates among existing bacterial strains (Cillóniz et al., 2018). In the absence of new generations of antibiotic drugs, the appropriate use of existing antibiotics is crucial to ensure the long-term availability of effective treatments for bacterial infections (Kaplan and Laing, 2004). Since the development of new antimicrobials is unsatisfactory, it is crucial to use the existing drugs appropriately.

Reducing the use of antibiotics results in lower antibiotic resistance. A landmark Finnish study on macrolide-resistant *Streptococcus pyogenes* demonstrated that decreasing macrolide consumption can diminish antimicrobial resistance. The research indicated that antibiotic resistance dropped from 9.2% in 1997 to 7.4% in 2000 (Seppälä et al., 1997). Several studies have demonstrated that reducing antibiotic use can lead to a decrease in bacterial resistance. For example, in the UK, a reduction in the incidence of antibiotic resistance was observed in local communities following a decrease in antibiotic prescriptions for urinary tract infections at the general practice level (Lewis, 2008). In Finland, a decrease in resistance levels of group A streptococci to erythromycin was observed following a nationwide reduction in the consumption of macrolide antibiotics (Seppälä et al., 1997). Australia has the seventh lowest rate of *E. coli* resistance to fluoroquinolones compared to European countries, where resistance rates are much higher. This lower resistance rate in Australia is largely due to restrictions imposed by the Pharmaceutical Benefits Scheme (PBS), which limits the prescribing of these medicines (Australian Commission on Safety and Quality in Health Care ACSQHC, 2016). It has to be clearly understood that, it is not only the misuse of antimicrobials that causes AMR; even their proper use can contribute to AMR. In fact, rational use of antimicrobials decreases the emergence of resistance but does not completely prevent it. Therefore, antimicrobials should be used only when truly necessary.

##### Strengthening good microbiology practices

Accurate specimen collection, handling, and prompt reporting using standard microbiology practices are crucial for preventing the spread of antimicrobial resistance AMR. Adhering to international testing standards, reporting resistance patterns to infection prevention and control (IPC) teams, and monitoring sterilization and disinfection activities are fundamental aspects of good microbiology practices (Prevention of hospital, 2002; National Policy for Containment of Antimicrobial Resistance, 2011).

##### Diagnostic Advancements

Early access to information about bacterial pathogens and their susceptibility facilitates targeted antimicrobial therapy and helps shorten the duration of treatment (Leekha et al., 2011). Traditional methods for identifying bacterial pathogens and performing susceptibility tests typically take at least 48 h. Delays in diagnostic procedures often lead to prolonged use of unsuitable empirical antibacterial therapies. However, new techniques such as susceptibility testing, DNA amplification assays, and advanced technologies like matrix-assisted laser desorption ionization time-of-flight mass spectrometry (MALDI-TOF), antimicrobial susceptibility testing, and PhenoTest BC enable faster and more precise pathogen identification. These techniques significantly enhance diagnostic accuracy and reduce time to results, which is crucial for effective antimicrobial stewardship (Maurer et al., 2017). The advantages of phenotypic antimicrobial susceptibility testing over genotypic testing are: (i) it allows for the prediction of both drug resistance and drug susceptibility, and (ii) it enables the quantification of a bacterial isolate’s susceptibility (Gajic et al., 2022).

#### Next-generation antimicrobial therapies and novel strategies to control AMR

To extend the life and efficacy of current antibiotics, novel strategies should focus on increasing awareness of antibiotic stewardship among all healthcare communities, enhancing research and development facilities to improve antibiotic production (Prestinaci et al., 2015) and prolonging the lifespan and effectiveness of currently available antibiotics, other approaches under investigation include whole genome sequencing, quorum quenching (QQ), viral phage therapy, monoclonal antibodies, drug repurposing, novel small-molecule antibiotics with a focus on biologics and non-antibiotic adjuvants, as well as complementary and alternative therapies (Uddin et al., 2021).

Over 80% (10 out of 12) of newly approved antibiotics belong to existing classes where resistance mechanisms are already known. Since the last report, only one new antibacterial, cefiderocol, has been approved. Cefiderocol is effective against all three critical Gram-negative bacteria, including those with various β-lactamases, such as ESBL and AmpC. Overall, the current clinical pipeline and recently approved antibacterial agents are insufficient to address the growing challenge of AMR (Murray et al., 2022). In addition to novel antimicrobials, vaccines have long been used to prevent infectious diseases and are essential for reducing the demand for antimicrobial drugs, which helps combat AMR. Unlike antibiotics, vaccines are not associated with the development of resistance. Therefore, the development and use of vaccines targeting antimicrobial-resistant bacterial infections, especially carbapenem-resistant *Enterobacterales* and *A. baumannii*, are crucial and could serve as a key strategy in fighting AMR transmission (WHO; WHO. Infection Prevention and Control; European strategic action plan on antibiotic resistance, 2011).

##### Adjuvants

To counteract the mechanisms of antibiotic resistance, β-lactamase inhibitors, efflux pump inhibitors, and outer membrane permeabilizers have been employed (). Using antibiotic adjuvants in combination with antibiotics has proven to be the most successful and effective strategy (González-Bello, 2017). Despite advances in β-lactamase inhibitors, there is an urgent need to develop effective inhibitors for class B β-lactamases (metallo-β-lactamases). Currently, there are no available inhibitors for these enzymes, and their spread among significant Gram-negative bacteria, such as Enterobacteriaceae, *P. aeruginosa*, *K. pneumoniae*, *E. coli*, and *A. baumannii*, is increasing dramatically (Palzkill, 2013). Adjuvants can effectively enhance the potency of existing antibiotics by lowering the minimum inhibitory concentration needed to kill bacteria. This helps preserve currently available treatment options (Melander and Melander, 2017; Laws et al., 2019).

A classic example is Augmentin^®^, which combines amoxicillin (a β-lactam antibiotic) with clavulanic acid (a β-lactamase inhibitor). Clavulanic acid enhances the efficacy of amoxicillin by inhibiting the β-lactamase enzyme that would otherwise inactivate the antibiotic (Worthington and Melander, 2013). This approach helps delay the onset of resistance, but not all β-lactamase enzymes produced by microorganisms are susceptible to β-lactamase inhibitors (Pulingam et al., 2022). In addition, scientists are creating new generations of β-lactamase inhibitors, such as BLI-489 and LK-157, which have demonstrated promising *in vitro* results against extended-spectrum β-lactamase (ESBL) microorganisms (Worthington and Melander, 2013).

The effectiveness of avibactam when combined with ceftazidime against *P. aeruginosa* is likely due to its potent inhibition of AmpC hydrolytic activity. This inhibition helps to counteract the resistance mechanisms that otherwise reduce the efficacy of ceftazidime (Levasseur et al., 2012). Moreover, Sixty-nine non-antibiotic compounds have been found to enhance the activity of minocycline against various microorganisms, including Methicillin-resistant *S. aureus* (MRSA) and several multidrug-resistant (MDR) species such as *P. aeruginosa* (Ejim et al., 2011; Worthington and Melander, 2013).

##### Efflux-pump inhibitors (EPIs)

EPIs are substances that block the action of efflux pumps, which are mechanisms used by bacteria to expel antibiotics and other substances. These efflux pumps are critical drug targets for developing combination strategies that use antibiotic efflux inhibitors to enhance the effectiveness of antibiotics (Lomovskaya and Watkins, 2001; Mahamoud et al., 2007). EPIs are typically simple, robust, and cost-effective chemicals that are generally well tolerated by humans (Marquez, 2005). Some EPIs also have the ability to inhibit bacterial biofilm formation. Compounds such as thioridazine, Phe-Arg β-naphthylamide (PAβN), and arylpiperazine NMP are categorized as efflux pump inhibitors. Their addition has been observed to significantly reduce biofilm formation in various bacteria, including *E. coli*, *K. pneumoniae*, *S. aureus*, and *Pseudomonas putida* (Kvist et al., 2008).

##### Combination of antimicrobials

Combination therapy of antibiotics has proven to be an effective strategy for restoring bacterial susceptibility. By combining two or more agents based on the susceptibility patterns of the infectious microbes, a synergistic effect can enhance treatment efficacy. This approach works by inhibiting targets in different pathways (e.g., antibiotics used in antituberculosis therapy), inhibiting distinct targets in the same pathway (e.g., the combination of trimethoprim and sulfamethoxazole), and inhibiting the exact same target through different mechanisms (e.g., the use of streptogramins) (Fischbach, 2011; Worthington and Melander, 2013). In the treatment of various infectious diseases, such as HIV/AIDS, malaria, and tuberculosis, combination antibiotic therapy is often preferred to enhance treatment efficacy (Worthington and Melander, 2013; Kerantzas and Jacobs, 2017).

Adverse effects from drug combinations can arise from both pharmacokinetic and pharmacodynamic interactions. Pharmacodynamic interactions occur when drugs directly influence each other’s actions, potentially amplifying their effects (synergistic) or diminishing them (antagonistic) (Coates et al., 2020). Additionally, pharmacodynamic effects can impact more than just the target bacteria, potentially causing unintended consequences in other parts of the body. On the other hand, pharmacokinetic interactions affect how drugs are absorbed, distributed, metabolized, and eliminated, which can alter their effective concentrations in the blood and tissues (Sinel et al., 2017).

### Quorum Sensing (QS) as Target to Control Biofilm Infection

#### QS inhibitors and anti-QS peptides

Microbes need to sense their surroundings and adjust their physiological processes to adapt and improve their survival. QS has been shown to play a crucial role in this adaptation, serving as a key regulatory mechanism in both bacteria and fungi (Dubern and Diggle, 2008). QS helps bacteria coordinate the production of virulence factors and establish infections. Recently, there has been growing interest in targeting nucleotide signaling as well. Nucleotides act as second messengers in this process, with cyclic diguanosine monophosphate (c-di-GMP), cyclic diadenosine monophosphate (c-di-AMP), cyclic guanosine monophosphate (cGMP), cyclic adenosine monophosphate (cAMP), and guanosine tetraphosphate (ppGpp) being key players (Kalia et al., 2013). Among the nucleotides mentioned, cyclic diguanosine monophosphate (c-di-GMP) has garnered significant attention due to its crucial role in biofilm formation in Gram-negative bacteria. Thus, targeting quorum sensing (QS) and modulating cyclic diguanosine monophosphate (c-di-GMP) are key objectives in the development of new anti-biofilm drugs (Wu et al., 2015).

Brackman et al. (Brackman et al., 2011). demonstrated that QS inhibitor increased the susceptibilities of both Gram-positive and - negative bacterial biofilms to antibiotics *in vitro* and *in vivo*. Palys’ group identified inhibitors of diguanylate cyclase (DGC), the enzyme responsible for synthesizing c-di-GMP. They discovered four small molecules that act as DGC antagonists and demonstrated effectiveness in disrupting biofilms formed by *P. aeruginosa* and *A. baumannii*. These molecules were able to disperse and inhibit biofilms of *P. aeruginosa* on urinary catheters. Notably, two of the molecules showed no toxic effects on eukaryotic cells, suggesting promising potential for controlling biofilm infections (Sambanthamoorthy et al., 2014).

One QS inhibitor, named ‘HAM,’ operates by targeting the thrombin receptor activated peptides (TraP) receptor, thereby disrupting bacterial communication. This inhibitor was tested for its effect on *S. aureus* biofilms and demonstrated an increased susceptibility of these biofilms to various antibiotic classes, highlighting its potential effectiveness. Furthermore, when HAM was tested against strains with mutations in genes involved in quorum sensing, only strains without mutations were affected. This suggests that HAM influences biofilm susceptibility specifically through the quorum-sensing system of *S. aureus* (Brackman et al., 2016).

The RNAIII-inhibiting peptide has been shown to inhibit staphylococcal TRAP/agr systems and reduce biofilm formation *in vivo*. These findings highlight the critical role of quorum sensing in biofilm infections within the host. In rat studies, RNAIII-inhibiting peptide effectively prevented methicillin-resistant *S. aureus* graft infections, indicating its potential as both an anti-quorum sensing and anti-biofilm agent (Balaban et al., 2007; LoVetri and Madhyastha, 2010).

### Disruption of bacterial amyloids to control bacterial biofilms

Many bacteria form functional amyloid fibers on their surfaces, which are crucial for biofilm formation and other community behaviors. Curli are extracellular amyloid fibers produced by *E. coli* and other Enterobacteriaceae. Two analogs of FN075 and BibC6, which are ring-fused 2-pyridones that target crucial protein–protein interactions involved in macromolecular assembly, have been found to inhibit curli biogenesis in *E. coli*. Pre-treatment with FN075 notably reduced virulence in a mouse model of urinary tract infection. While curli and type 1 pili each play distinct and independent roles in promoting *E. coli* biofilms, FN075s ability to inhibit the formation of both structures makes it a promising compound for antibiofilm and anti-virulence applications (Cegelski et al., 2009).

Bacterial amyloids have emerged as a significant area of research. These structures, found in both bacteria and fungi, play a crucial role in the formation of biofilms by allowing bacteria to adhere to each other and to host surfaces. Targeting and disrupting amyloid structures could offer a novel approach to controlling bacterial biofilms (Romero et al., 2010). Recent studies have demonstrated that the formation of amyloid-like fibers in *B. subtilis* biofilms can be inhibited using two specific molecules: AA-861, a benzoquinone derivative, and parthenolide, a sesquiterpene lactone. These molecules were identified from a broad screening of bioactive compounds. AA-861 was found to block the formation of functional amyloid-like fibers by the TasA protein, while parthenolide disrupted pre-existing biofilms. Both compounds also effectively prevented the formation of biofilms in other bacterial species known to secrete amyloid proteins (Romero et al., 2013).

#### Phage therapy

Phage therapy involves using bacterial viruses, or bacteriophages, to target and kill pathogenic bacteria. Often referred to as “bacteria eaters,” phages offer several advantages over traditional antibiotics, including their easy availability, diversity, ability to increase in number spontaneously, low inherent toxicity, specific host range, lack of cross-resistance with antibiotics, and minimal environmental impact. However, challenges remain, such as selecting the appropriate phages, addressing their narrow host range, developing effective formulations, managing potential immune reactions, and ensuring clinician understanding. While phage therapy is not likely to replace antibiotics entirely, it has proven effective in treating topical infections where antibiotics have failed (Brives and Pourraz, 2020).

Bacteriophages are being explored as a potential solution to combat antibiotic-resistant microorganisms. For instance, Phico Therapeutics is developing SASPject™, a technology designed to selectively target and destroy specific bacteria—achieving up to 99.9% bacterial elimination within 2 min while sparing the normal flora. SASPject™ utilizes bacteriophages, which are modified and disabled bacterial viruses carrying genes for antibacterial proteins. Once these genes are introduced into the target bacteria, they lead to the inactivation of bacterial DNA, thus neutralizing the infection (Fairhead, 2009). Combining phages with antibiotics has shown promising results, particularly in tackling multidrug-resistant biofilms. This approach leverages the unique mechanisms of both therapies to enhance bacterial eradication, with phages targeting specific bacteria and antibiotics addressing a broader spectrum. This synergy can be especially effective against complex infections where traditional treatments have failed (Akturk et al., 2019). Phage therapeutic approaches can also serve as adjunctive therapies to enhance the effectiveness of antibiotic treatments. They can work alongside probiotic supplements and beneficial microflora, potentially improving overall treatment outcomes and maintaining a healthy balance of microbial communities (Jassim and Limoges, 2014). In particular, pre-treating biofilms with phages has been demonstrated to enhance the effectiveness of antibiotics. This approach can make the biofilms more susceptible to antibiotic treatment, potentially improving treatment outcomes for infections caused by resistant bacteria (Townsend et al., 2020).

##### Optimizing drug delivery systems

To address the limited cell permeability of antibiotics, promising delivery systems have been developed to enhance the drug’s ability to enter the cell (Li et al., 2023). A crucial strategy for overcoming antibiotic resistance is to better utilize transport systems, such as synthetic siderophore derivatives that enhance antibiotic entry. A notable study demonstrated that a conjugate containing ampicillin achieved remarkable results, showing a 100-fold increase in efficacy against Gram-negative enterobacteria compared to ampicillin alone, and a 1000-fold increase in inhibiting the growth of *P. aeruginosa* (Mollmann et al., 2009).

The use of nanoparticles (NPs) appears to be an effective strategy for addressing AMR by functioning as either drug delivery systems (DDS) or active antibacterial agents. When employed as drug nanocarriers, NPs can help overcome bacterial resistance by shielding the loaded antibiotics from biological degradation and inhibiting efflux pumps (Dey et al., 2022). Additionally, NPs enable controlled and sustained drug release, maintaining active doses of therapeutic agents for extended periods. As a result, a lower dose of the antibacterial agent is needed to achieve a therapeutic effect, which helps minimize side effects on healthy cells and tissues (Chamundeeswari et al., 2019). In addition to functioning as DDS, NPs also have intrinsic antibacterial activity, making them potent therapeutic agents for antibacterial therapy. Certain types of NPs, such as metallic nanoparticles, can generate reactive oxygen species (ROS) and reactive nitric oxide (NO) that damage bacterial cellular components. Furthermore, NPs can inhibit DNA and enzyme synthesis, disrupt energy transduction by affecting the electron transport chain in the transmembrane, and release heavy metal ions that have harmful effects on bacteria (Alabdali et al., 2022).

Polymeric nanoparticles have emerged as a promising solution to various challenges in antimicrobial therapy. One approach involves using hydrophilic polymersomes containing encapsulated vancomycin, which improves treatment efficacy, particularly against MRSA infections. Nano-emulsification systems enhance drug solubilization in water and increase bioavailability by creating smaller particles that expand the surface area for absorption. This system facilitates better drug dispersion, leading to increased solubility and more efficient absorption in the body. Additionally, a self-nano-emulsifying preconcentrate (EB-P) of ebselen has been developed, demonstrating more potent antifungal activity against azole-resistant *Candida* albicans strains (Vartak et al., 2020; Menon et al., 2021). Combining nanoparticles with targeting-based strategies offers a potential approach for delivering high concentrations of antimicrobial agents directly to the infection site, while minimizing toxicity to non-target cells (Huang et al., 2021).

Moreover, liposome lipid bilayers can directly interact with or fuse with bacterial cell walls, enhancing the concentration of antibiotics within the bacteria and thereby improving the therapeutic effect of the loaded antibiotic (Drulis-Kawa and Dorotkiewicz-Jach, 2010). Additionally, liposome-encapsulated antibiotics have demonstrated the ability to overcome various resistance mechanisms of microorganisms, such as impermeable outer membranes, efflux mechanisms, and enzymatic degradation. In conclusion, due to their unique physicochemical properties and benefits as antibiotic carriers, liposomes represent a highly promising strategy for restoring treatment options against bacterial infections that are currently untreatable (Ferreira et al., 2021).

#### Optimization of dosing regimens

Dosing regimens and durations of antibiotic treatments should be optimized to strike a balance: they need to be sufficiently high to maximize the antibacterial effect while being as brief as possible to reduce the risk of developing resistance (Negri et al., 1994; Baquero and Negri, 1997; Guillemot et al., 1998). A key component of antimicrobial stewardship is the optimization of antimicrobial dosing, which involves considering individual patient factors (such as age, renal function, and weight), the causative organism, the infection site (e.g., endocarditis, meningitis, or osteomyelitis), and the drug’s pharmacokinetic and pharmacodynamic properties. Practical applications of these principles include prolonged or continuous infusion of β-lactams, extended-interval dosing of aminoglycosides, and adjusted dosing of fluoroquinolones for *S. pneumoniae* in community-acquired pneumonia and for *Pseudomonas* in hospital-acquired pneumonia (HAP) and ventilator-associated pneumonia (VAP) (MaHAaCA, 2018).

#### Utility of artificial intelligence (AI)

Utilizing AI algorithms offers a promising approach to accelerate the drug discovery process. Researchers used a neural network to screen approximately 7,500 molecules, which led to the discovery of a compound named abaucin. This compound has shown effectiveness in treating *A. baumannii* infections (Liu et al., 2023). AI, especially through machine learning (ML) and deep learning (DL) techniques, is significantly advancing both the design of new antibiotics and the development of drug combinations. ML and DL algorithms analyze extensive biological and chemical data to identify potential antibiotic candidates and predict synergistic interactions between different drugs. This approach aids in discovering effective drug combinations that can improve treatment efficacy, particularly against resistant pathogens, and in optimizing existing drugs for enhanced therapeutic outcomes (Hu et al., 2019). Machine learning algorithms play a crucial role in analyzing patterns of AMR. By analyzing extensive datasets of bacterial and fungal genetic information, these algorithms can predict resistance patterns and identify emerging resistant strains. This capability helps healthcare providers and policymakers make informed decisions by forecasting which pathogens are likely to develop resistance to specific drugs or compounds. As a result, these predictions can guide more effective treatment strategies and inform policies to manage and mitigate AMR (Rabaan et al., 2022; Amsterdam, 2023).

Machine-learning models are becoming increasingly valuable for AMR surveillance. By analyzing data on antimicrobial usage and resistance patterns, these models assist public health authorities in monitoring and predicting emerging resistance trends. They enable timely responses to potential outbreaks by identifying at-risk populations and areas. This proactive approach supports informed decision-making and enhances preparedness and response strategies, ultimately aiding in the management and containment of AMR issues (Rabaan et al., 2022). These applications help reduce the overall burden of AMR (Ali et al., 2023).

## Conclusion

Antibacterial resistance represents a multifaceted and evolving challenge that threatens the efficacy of antibacterial therapy worldwide. This review has explored the diverse types and mechanisms of antibacterial resistance, shedding light on the intricate strategies employed by bacteria to evade the effects of antimicrobial agents. From enzymatic degradation and decreased accumulation, target alteration to biofilm formation and HGT, bacteria have developed a myriad of methods to withstand antibacterial pressure. Understanding these resistance mechanisms is paramount for the development of novel treatment strategies and the implementation of effective infection control measures. Moreover, it discusses the interventions taken and next-generation antimicrobials to contain AMR.

## Abbreviations

AMEs, Aminoglycoside-modifying enzymes; AMR, Antimicrobial resistance; CATs, chloramphenicol acetyltransferases; DHFR, dihydrofolate reductase; DHPS, dihydropteroate synthase; ESBLs, Extended-Spectrum β-Lactamases; EPS, extracellular polymeric substance; HGT, horizontal gene transfer; MIC, minimum inhibitory concentration; LPS, lipopolysaccharides; MDR, Multidrug resistance; PBP, Penicillin-Binding Proteins; QRDR, quinolone-resistance-determining region; VRSA, vancomycin resistance in *Staphylococcus aureus.*
